# Bone Sarcomas: From Biology to Targeted Therapies

**DOI:** 10.1155/2012/301975

**Published:** 2012-11-27

**Authors:** Nathalie Gaspar, Angela Di Giannatale, Birgit Geoerger, Françoise Redini, Nadège Corradini, Natacha Enz-Werle, Franck Tirode, Perrine Marec-Berard, Jean-Claude Gentet, Valérie Laurence, Sophie Piperno-Neumann, Odile Oberlin, Laurence Brugieres

**Affiliations:** ^1^Department of Oncology for Children and Adolescents, Institut Gustave Roussy, 114 rue Edouard Vaillant, 94805 Villejuif Cedex, France; ^2^Inserm U957-EA 3822, Faculté de Médecine, 1 rue Gaston Veil, 44035 Nantes Cedex 1, France; ^3^Oncopediatric Departement, CHU de Nantes, Boulevard Jacques Monod, 44093 Nantes, France; ^4^Pediatric Onco-Hematology Department, EA 4438UdS, CHRU Strasbourg, Avenue Molière, 67000 Strasbourg, France; ^5^Unité 830 INSERM, Institut Curie, Centre de Recherche, 26 rue d'Ulm, 75248 Paris Cedex 05, France; ^6^Oncopediatric Departement, Centre Léon Bérard, 28 rue Laennec, 69008 Lyon, France; ^7^Oncopediatric Departement, Hôpital La Timone, 264 rue Saint Pierre, 13385 Marseille Cedex 5, France; ^8^Department of Medical Oncology, Institut Curie, 26 rue d'Ulm, 75248 Paris Cedex 05, France

## Abstract

Primary malignant bone tumours, osteosarcomas, and Ewing sarcomas are rare diseases which occur mainly in adolescents and young adults. With the current therapies, some patients remain very difficult to treat, such as tumour with poor histological response to preoperative CT (or large initial tumour volume for Ewing sarcomas not operated), patients with multiple metastases at or those who relapsed. In order to develop new therapies against these rare tumours, we need to unveil the key driving factors and molecular abnormalities behind the malignant characteristics and to broaden our understanding of the phenomena sustaining the metastatic phenotype and treatment resistance in these tumours. In this paper, starting with the biology of these tumours, we will discuss potential therapeutic targets aimed at increasing local tumour control, limiting metastatic spread, and finally improving patient survival.

## 1. Introduction

Primary bone sarcomas, osteosarcomas (OS), and Ewing sarcomas (EW) are diseases occurring mainly in adolescent and young adults and account for around 15% of childhood/adolescent cancers. First-line therapeutic strategies in these diseases consisted in chemotherapy (CT) before and after local treatment (including high-dose CT for high-risk EW [1]) and a local treatment by surgery but also or only by radiotherapy in EW. Some patients remain very difficult to treat, such as tumour with poor histological response to preoperative CT (or large initial tumour volume for EW not operated) [2, 3], patients with multiple metastases at diagnostic [1, 4], or those who relapsed [5].

In order to develop new therapies against these diseases we need to unveil the founder molecular abnormalities underlying the malignant characteristics and to broaden our understanding of the phenomena sustaining the metastatic phenotype and treatment resistance in these tumours. Both diseases are sustained by different biology abnormalities but also share some common characteristics (angiogenesis, etc.).

**Figure 1:**
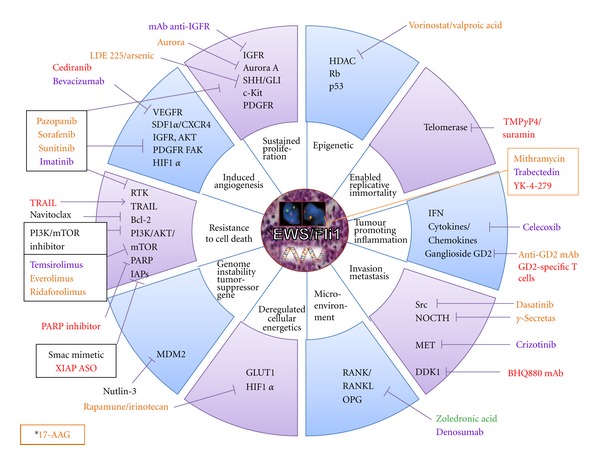
Targets and therapies in preclinical and clinical development in children and adolescent bone sarcomas. (A) Ewing sarcomas. (B) Osteosarcomas. The different colors described the current clinical development of the drugs. (Red) Preclinical: EW and OS; (Orange) Phase I: all paediatric studies; (Blue) Phase II: specific EW, OS, bone tumours; (Green) Phase III: specific EW and/or OS; (Black) Phase I or II in adults: all solid tumours. *17-AAG is an HSP90 inhibitor which targets client proteins involved in all tumour characteristics.

**Figure 2:**
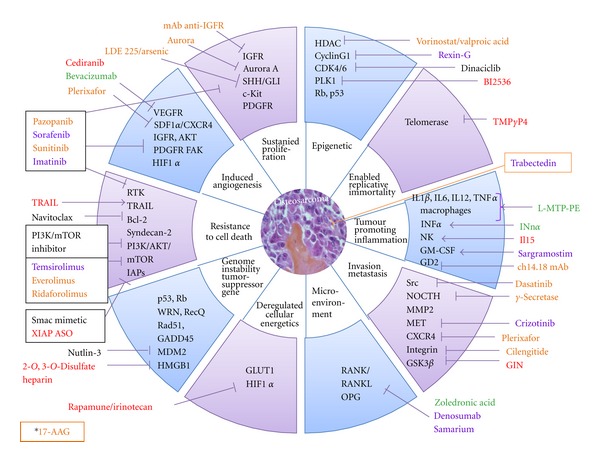


The main objective of this paper is to discuss potential therapeutic targets aimed at increasing local control of the primary tumour, limiting metastatic spread, and finally improving patient survival. We then review preclinical data and both paediatric and adult trials performed or ongoing and choose to present them by pathway rather than by tumour.  Table 1 and Figures 1 and 2 present the same data by tumour type.

## 2. Biology of Bone Tumours

### 2.1. Biology of Ewing Sarcoma: A Cell of Mesenchymal Origin Driven by an Aberrant Fusion Protein, EWS-Ets

EW is characterised by a group of translocations which oppose a gene from the EWS family with a gene from the ETS family arising in cells of mesenchymal origin [6]. The most frequent translocation is t(11;22). It leads to an aberrant fusion protein which is responsible for the malignant phenotype [7]. EWS-Ets is a transcription factor with a DNA binding domain (Ets; FLI1 in 85%) and a transcription enhancer domain (EWS) [8]. The altered intrinsic EWS-FLI1 region facilitates the formation of protein-protein interactions that regulate the transcription of numerous genes and mRNA alternative splicing [8]. Numerous biological pathways are modulated by EWS-FLI1 activity: IGFR, PDGFR, VEGFR, SHH pathway activation; Wnt, TGF*β*RII pathway inhibition, and lead to the EW malignant phenotype: proliferation, angiogenesis, immune system escape, metastatic potential, and treatment resistance [8].

### 2.2. Biology of Osteosarcoma: Osteoblast or Cells of Mesenchymal Origin with a Complex Biology Producing Osteoid Matrix

OS is a malignant tumour that produces osteoid tissue. Different mesenchymal components found in different OS subtypes suggest that OS arise from a more pluripotent cell than the osteoblast.

OS belong to the spectrum of genetic predisposition to cancer syndromes (Li Fraumeni, hereditary retinoblastoma, Rothmund-Thomson, Werner syndromes). Molecular abnormalities influence various tumour characteristics and may be implicated in several biological pathways: sustaining proliferative signalling (IGFR, SHH/GLI, PDGFR, c-KIT), evading cell growth suppressors (p53, RB, CDK), resisting cell death (ERK activation, proapoptotic molecule inhibition, antiapoptotic molecule activation Bcl2, syndecan-2, autophagy inhibition), enabling replicative immortality (telomerase), increasing angiogenesis (VEGFR, IGFR, PDGFR, HIF1*α*), and activating invasion and metastasis, genome instability (p53, Rad51, GADD45), evading immune destruction (IFN), reprogramming energy metabolism and hypoxic driven therapeutic resistance (HIF1*α*, GLUT1), and interacting with the bone microenvironment (RANK/RANKL/OPG).

## 3. Therapeutic Options for Bone Sarcomas

### 3.1. EWS-FLI1 Inhibition in Ewing Sarcomas

The fusion protein EWS-FLI1, exclusively expressed in EW tumour cells, is an ideal target for specifically treating EW without affecting normal cells.

Decreased EWS-FLI1 expression by antisense oligonucleotides [9] or RNA [10], small interference RNA (siRNA) through nanoparticles [11], inhibits cell proliferation and tumour growth of EW xenografts. The pharmacological delivery of these large molecules in patients is not yet solved. Mithramycin has been identified by high-throughput screening as another inhibitor of the EWS-FLI1 oncogenic transcription factor and has shown *in vitro* and *in vivo* activity against EW [12]. Mithramycin is currently being tested at the NCI against EW in children and adults (NCT01601570).

An alternative strategy is to target the interaction between EWS-FLI1 and its partner proteins in the transcriptional complexes in order to inhibit EWS-FLI1 function. YK-4-279 inhibits EWS-FLI1/RNA helicase A (RHA) interaction and induces apoptosis and tumour regression in EW models [13].

 Trabectedin is an alkylating agent with increased efficacy in EW compared to other paediatric sarcomas (e.g., OS; rhabdomyosarcoma) through EWS-FLI1 inhibition [14, 15]. However, in children/adolescents, compassionate use of trabectedin and phases I/II trials yielded only one complete response (CR) lasting 6 months and stable diseases (SD) in 5 EW [14, 16–18]. In OS, only 2 partial responses (PR) out of 27 treated patients were observed. Tolerance in paediatric phases I/II trials [14, 16] was acceptable (thrombocytopenia, reversible hepatic toxicity).

Combined inhibition of EWS-FLI1 (oligonucleotide) and EWS-FLI1-modulated pathways (e.g., mTOR) increased the antitumour effect (apoptosis, *in vivo* tumour regression) [19].

### 3.2. Inhibition of Growth Factor Signalling Pathways

Most of the signalling pathways are involved in cell proliferation and resistance to apoptosis. They are mediated by proteins with kinase activity (tyrosine TK or serine SK kinases), located on the tumour cell surface, in the cytoplasm, or the nucleus. These proteins could be inhibited by two different approaches: monoclonal antibodies directed against extramembrane receptor and small molecule inhibitors of the intracellular kinase domain.

#### 3.2.1. The IGF-1R/PI3K/AKT/mTOR Pathway

The IGF-1R pathway plays an important role in paediatric cancers, including OS/EW [20]. Both tumours have a peak incidence at puberty, and OS occur in an area of a high bone growth rate at long bone metaphyses, suggesting a role of growth hormone and IGF-1. Like others, the IGF-1R pathway activates downstream pathways PI3K/Akt/mTOR and stimulates OS/EW cell survival and angiogenesis through HIF-1*α* and VEGF secretion.

With different anti-IGF-1R monoclonal antibodies, children/adolescents suffering from relapsed/refractory EW achieved SD in phase I trials [21] and an objective response rate of 10–15% in paediatric/adult phase II trials [22–24]. SD was observed in relapsed/refractory OS patients (SCH 717454, P04720, unpublished data, NCT00617890) [25].

Predictive factors of response remain insufficiently known. Reduced activity in an IGF system might be associated with tumour progression and poor response to treatment [26], high expression levels of IGF-IR, IR, and IGF-I mRNAs with increased survival, and high circulating IGF-1 levels with a low risk of progression [27].

Unfortunately, the median duration of EW response was only 5–7 months [22, 23], probably because tumour cells escape IGF-1R inhibition, through AKT or through activation of other signalling pathways (e.g., other TK receptors, mTOR) [28]. These observations have prompted clinical researchers to consider using either a combination of monotargeted inhibitors or multitargeted inhibitors.

Rapamycin, the mTOR inhibitor, was first used in children to prevent graft rejection. mTOR is an intracytoplasmic SK regulated by AKT. In OS cells, rapamycin inhibits proliferation through ezrin [29], a protein involved in intracellular signal transduction and migration [30]. In paediatric EW, phospho-mTOR overexpression is correlated with survival [31]. Paediatric phase I trials of everolimus [32] and temsirolimus [33] have demonstrated a good tolerance profile. One OS patient treated with everolimus achieved prolonged SD out of 5 patients treated with mTOR inhibitors [32]. The phase II trial of ridaforolimus in advanced bone and soft tissue sarcomas obtained a low response rate <2% (2/4 responders had OS), but 28% obtained a clinical benefit [34]. A double blind phase III maintenance trial comparing ridaforolimus and placebo (SUCCEED trial) in advanced bone and soft tissue sarcoma after stabilisation or response with CT has included 50 bone sarcoma patients showing an increased progression free survival (PFS) in patients treated with ridaforolimus [35]. A paediatric phase II is ongoing in refractory/relapsed OS, in Brazil (NCT01216826). All these mTOR inhibitors inhibit TORC1. However, two mTOR complexes participate in two functionally disparate protein complexes, TORC1 and TORC2, both being associated with oncogenesis. TORC2 and subsequent AKT activation is suggested to induce resistance to TORC1 inhibition, and the dual TORC1/TORC2 small molecule inhibitor is being developed in adults (OSI-027, NCT00698243).

Strategies targeting the IGF-1R/PI3K/AKT/mTOR pathway simultaneously at several levels are being evaluated. An adult phase I combination of the anti-IGF1-R antibody cixutumumab and temsirolimus showed good tolerance and tumour regression of more than 20% in 5/17 (29%) EW patients who remained on study for 8 to 27 months, with a CR in 1/6 of EW patients who previously developed resistance to a different IGF-1R inhibitor antibody [36]. The phase II in younger patient with refractory (1–30 tears) or relapsed sarcomas is ongoing (NCT01614795) in USA. A phase I-II trial of ridaforolimus combined with the anti-IGF1R antibody Dalotuzumab is ongoing (NCT01431547) in children in Europe and USA. Dual PI3K/mTOR inhibitors are being tested in an adult phase I trial and a dual mTOR/DNA-PK inhibitor (CC-115) in an adolescent/adult phase I trial (NCT01353625).

#### 3.2.2. Multitarget Inhibitors

Imatinib mesylate inhibits PDGFR, c-KIT, and BCR-ABL. High expression of c-KIT and PDGFR is observed in EW/OS [37] and associated with low EFS but not with poor response to CT [37]. Imatinib appeared to exhibit anti-EW activity *in vitro* and in xenografts [38]. Expression of imatinib targets is not sufficient to confer drug sensitivity [39]. Several phase II trials have shown some stabilisation of bone sarcomas (3/20 EW, 7/26 OS) with a median PFS <2 months [40, 41]. In a COG paediatric phase II trial, only 1/24 EW achieved a PR [42]. Preclinical data showed increased antitumour activity of imatinib when combined with doxorubicin and vincristine [43] in EW or ifosfamide in OS. 

Dasatinib which inhibits Src and BCR-ABL shows *in vitro* cytostatic and antimigration effects and no apoptosis in EW [44]. Src plays a role in OS cell adhesion/migration through a decrease in FAK, but its inhibition does not prevent metastasis [45], suggesting that Src plays a secondary role in this process. A phase I paediatric trial showed similar dasatinib pharmacokinetics in children and adults [46].

Sorafenib inhibits BRaf, c-KIT, PDGFR, VEGFR, and RET. In OS, sorafenib inhibits proliferation of tumour growth, angiogenesis (VEGF), invasion (MMP2), and the emergence of pulmonary metastases (Erzin/*β*4-intégrin/PI3K) and induces apoptosis [47]. A phase II trial of 35 patients ≥14 years with OS under 2nd/3rd-line therapy achieved 14% of objective responses (3PR, 2MR) and 29% of tumour control (12 additional SD). Tumour control lasted ≥6 months for 8 patients. The median PFS and survival were 4 and 7 months, respectively [48]. 

Sunitinib inhibits Flt3, c-KIT, PDGFR, and VEGF. Efficacy was observed with *in vivo* models of most paediatric tumours, including 4/5 EW xenografts [49]. In a paediatric phase I trial, the main toxicities were haematological and cardiac for children previously treated with anthracyclines [50, 51].

Pazopanib inhibits VEGFR1–3, PDGFR*α*/*β*, and c-KIT. Pazopanib showed activity in paediatric *in vivo* tumour models when used as a single agent (EW, EFS [52]) or combined with metronomic topotecan (OS, tumour regression [53]). A phase II study of pazopanib in relapsed bone sarcomas is ready to begin in Europe. The phase I in children with solid tumours showed good tolerance [54]. The combination pazopanib/everolimus is currently being tested in an adult phase I (NCT01430572). Furthermore, there is increasing information that mTOR inhibition can reverse resistance to growth receptor inhibition in other solid tumours including breast cancer [55, 56]. 

#### 3.2.3. Cell Growth Inhibition Dependent on Cell Cycle Regulators

The CDK (cyclin-dependent kinase) inhibitor dinaciclib induces *in vitro* OS apoptosis [57]. The phase I/II trial of Rexin-G, a pathotrophic nanoparticle bearing a cytocidal cyclin G1 construct, in relapsed OS showed low toxicity, 2/3 SD, and survival lasting 7 months- [58]. Aurora A plays a crucial role during mitosis. The Aurora A inhibitor, MLN8237, led to prolonged CR in *in vivo* EW/OS models [59]. Two Aurora A inhibitors, MLN8237 (NCT01154816/NCT00739427) and AT9283 (NCT00985868/NCT01431664), are under development in paediatric phase I/II studies. The Polo-like kinase 1 (PLK1) selective inhibitor, BI 2536, exerted antiproliferative effects and induced mitotic death in OS cell lines [60].

MDM2 is an oncoprotein that negatively regulates p53 and is overexpressed in p53 wild-type cancers. The MDM2 inhibitor, nutlin-3, activates the p53 signalisation pathway leading to major tumour regressions in OS xenografts through apoptosis [61, 62]. This effect is also seen in p53 wild-type EW and can be increased by either NF-*κ*B inhibition [63] through TNF-alpha [64] or HDAC inhibitors [65]. An adult phase I of an oral MDM2 inhibitor (RO5503781) is ongoing in solid cancers (NCT01462175) and a study in sarcoma in preparation.

### 3.3. Resistance to Cell Death

Resistance to apoptosis is a key element in tumour progression and chemoresistance [66]. Its mechanisms are increased survival signals (growth factors/TK receptors, downstream pathways), overexpression of antiapoptotic molecules (Bcl-2, Bcl-XL, FAK in OS), underexpression of proapoptotic molecules (Bim in OS), or resistance to cell death receptors Fas/FasL (Fas ligand) or TRAIL. The BCL2 inhibitor, navitoclax, is developed in adult refractory tumours in combination with docetaxel. Toxicity is acceptable, and a few responses (2 PR, 5 SD) have been achieved [67]. TRAIL-induced apoptosis in murine models inhibits EW/OS tumour growth, decreases osteolysis, prolongs survival, and decreases lung metastases from OS [68]. Combining them with imatinib further increased TRAIL effect on tumour growth and metastases in *in vivo* EW models [69]. The fully human monoclonal antibody directed against DR5 (human death receptor 5), conatumumab, activates caspases, and induces apoptosis [70]. Phases I/II of conatumumab combined with the anti-IGF1R antibody AMG479 in advanced sarcomas showed only SD (1OS/1EW) [71] and combined with doxorubicin did not show advantages compared to doxorubicin alone in advanced soft-tissue sarcomas [72]. IAPs (inhibitor of apoptosis proteins) inhibit caspase-dependent apoptosis. Smac, a mitochondrial protein, binds to IAPs, impedes the formation of the protective complex IAP/caspase, and facilitates caspase degradation by the proteasome. The Smac mimetic, LCL161, increases survival of paediatric *in vivo* models, including 5/6 OS and glioblastomas [73]. The adult phase I trial of LCL161 in solid tumours (NCT01098838) has just been completed, and a combination trial with paclitaxel is ongoing (NCT01240655). The X-linked IAP antisense oligonucleotide (XIAP ASO-AEG35156) in paediatric tumour cell lines decreases XIAP in OS, RMS, and EW and sensitizes OS to doxorubicin, etoposide, and vincristine [74]. Poly(ADP-ribose) polymerase (PARP) inhibitors induce apoptosis and tumour CR in EW models, and EWS-FLI1 fusion genes maintain the expression of PARP1, a DNA damage response protein and transcriptional coregulator, thereby enforcing oncogene-dependent sensitivity to PARP-1 inhibition [75]. Inhibition of survivin induces apoptosis [76] and reverts CT resistance (etoposide, cisplatin, and doxorubicin) in OS cell lines [77].

Autophagy, a cell survival process implicated in tumourigenesis and chemoresistance [78], participates, through HMGB1, in OS resistance to doxorubicin, cisplatin, and methotrexate. HMGB1 inhibition by siRNA restores chemosensitivity [79]. HMGB1 binds to Beclin1, which regulates the formation of the Beclin1-PI3KC3 complex and promotes autophagy. The 2-*O*,3-*O*-disulfate heparin (ODSH) is a low molecular weight anticoagulant with anti-inflammatory activity but low anticoagulant activity [80]. It might exhibit an antitumour action through inhibition of heparinase (invasion), selectins (pulmonary metastatic spread), and RAGE II which is no longer able to bind to HMGB1 (proinflammatory and proautophagy roles).

Replicative immortality through the restoration of telomerase activity in cancer cells induces resistance to cell death. Telomerase activity is present in 85% of metastases (100% EW, 75% OS), but in only 12% of primary OS/EW tumours and associated with shortened telomeres and decreased patient survival [81]. The telomerase inhibitor, TMPyP4, inhibits telomerase enzyme activity, but inhibition of cell growth depends on the cellular context [82]. Telomerase activity is induced by EWS-FLI [83]. Telomerase is inhibited by suramin in OS [84] and imatinib [85], doxorubicin [86], or irradiation [87] in EW. 

#### 3.3.1. Inhibition of Angiogenesis and Hypoxia-Driven Resistance via mTOR Inhibition

Angiogenesis forms new capillaries from preexisting vessels, and vasculogenesis is the formation of new vessels from bone-marrow-derived progenitor cells [88]. PDGFR, VEGF, VEGFR and their downstream pathways (PI3K/AKT) are implicated in angiogenesis, VEGFR, and Notch (DLL4) in vasculogenesis, explaining the antiangiogenic effect of the multitargeted therapies described above. These receptors are overexpressed in OS/EW and associated with a poor prognosis [89, 90]. After cytotoxic CT, the number of bone marrow progenitor cells increases, promoting expansion of residual tumour cells or micrometastases [88]. Hypoxia increases these phenomena, especially through induction of HIF1*α* expression [91], a factor associated with increased OS/EW aggressiveness [92, 93] and metastatic potential. HIF1*α* expression is also induced by PI3K/AKT/mTOR, RAS/MAPK pathways, and calcium signalling. HIF1*α* plays an additional role in bone sarcoma cell proliferation and apoptosis [94] and modulates EWS-FLI expression in EW [92].

Bevacizumab is an anti-VEGF IgG1 monoclonal antibody which inhibits VEGF/VEGFR-1 and VEGFR-2 interactions and VEGF-dependent angiogenesis. Tolerance in children/adolescents is good with a few side effects (proteinuria, thrombotic risk). A randomised phase II trial of bevacizumab combined with vincristine/topotecan/cyclophosphamide in first recurrent EW showed good tolerance (COG-AEWS0521, NCT00516295). A phase II trial combining bevacizumab with CT (MAP/MAPIE: methotrexate/adriamycin/platinum/ifosfamide/etoposide) as 1st-line therapy in OS is ongoing (NCT00667342).

Cediranib which inhibits VEGFR delayed tumour growth in 3/3 EW and 4/5 OS (1 CR) in *in vivo* models [95]. This delay in tumour growth was further increased when cediranib was combined with rapamycin, an mTOR inhibitor but not when combined with CT (vincristine, cyclophosphamide, cisplatin) [96]. DLL4 inhibitors are being tested in phase I in adults (neutralising antibody REGN421, NCT00871559). SDF-1*α*/CXCR4 inhibition might also make it possible to target vasculogenesis, especially in tumours resistant to anti-VEGF therapies [88].

mTOR and topoisomerase I inhibitors decrease HIF-1*α* accumulation leading to a major antitumour effect mainly when combined [97]. An SFCE (*Société Française des Cancers de l'Enfant*) paediatric phase I trial (RAPIRI, NCT01282697) combining rapamycin/irinotecan is ongoing.

### 3.4. Inhibition of the Metastatic Phenotype

Each step of the metastatic process could be targeted by different therapeutic classes [98]. OS invasion of the host extracellular matrix depends on the Notch/Hes1 pathway [99]. Its inhibition by gamma secretase inhibitors prevents the formation of metastases and induces tumour regression [9]. In EW, Notch is involved in neural differentiation, proliferation, and apoptosis, but its inhibition in established tumour models yielded a poor antitumour effect [100]. Paediatric phase I trials with the gamma secretase inhibitors MK-0752 in leukemia and CNS tumours showed good tolerance [101, 102].

Migration and the passage in the systemic circulation depend on the Met/HGF pathway [103, 104]. The ALK/MET inhibitor, crizotinib (PF-2341066), decreased proliferation, survival, invasion, and clonogenicity *in vitro*, tumour growth, and osteolysis in *in vivo* OS models [103, 105, 106]. A phase II for patients ≥15 years is about to start in patients with MET or ALK-driven sarcoma and lymphomas (CREATE, NCT01524926).

Resistance to anoikis and the capacity to escape the immune system allow tumour cells to survive in the bloodstream. Anoikis is an apoptotic death induced by the loss of intercellular and cell/extracellular matrix contacts and depends on Src/PI3K/AKT and Wnt/*β*-catenin/NF-*κ*B pathways. In OS, GIN, the GSK3beta inhibitor stimulates the Wnt/*β*-catenin pathway and induces intranuclear passage of *β*-catenin [107]. A phase I of the LY2090314 (GSK3 inhibitor)/pemetrexed/carboplatin combination is ongoing in adults with progressive solid tumours, with good tolerance and restoration of *β*-catenin expression [108]. DDK1 inhibitors interfere with the Wnt pathway and bone metabolism. Adult phase I studies with monoclonal anti-DDK1 antibodies (LY2812176, NCT01457417; BHQ880, NCT00741377) are ongoing.

The arrival of circulating metastatic tumour cells in the lungs depends on chemokines and adhesion, then extravasation into target tissues depends on proteinases (MMP2, MMP9). CXCR4 is the main chemokine involved in OS [98]. CXCR4 inhibitors are used in humans to treat HIV infection and to mobilise hematopoietic stem cells (AMD3100, plerixafor). A paediatric phase I trial of plerixafor as chemosensitiser is ongoing in children with relapsed acute leukemia and myelodysplastic syndrome (NCT01319864). Adhesion and survival in the novel microenvironment depend on Erzin/*β*4-integrin/PI3K pathway and Fas/FasL-mediated resistance to apoptosis [109].

Dormancy is the prolonged survival in a quiescent state of isolated cells or micrometastases that might be responsible for late metastatic recurrences or resistance to cytotoxics. Dormancy depends on *α*v*β*1 integrin activation of NF-*κ*B, antiapoptotic molecule Bcl-XL, and the ERK/p38-MAPK ratio [110]. *β*4 and *β*3 integrins are expressed in OS and implicated in resistance to TNF*α*-dependent apoptosis [111, 112]. Their inactivation is sufficient to revert the metastatic phenotype, but not inactivation of *β*1 integrin. Cilengitide is the unique integrin inhibitor (high affinity selective antagonist of *α*v*β*3/*α*v*β*5) currently under development in children. It induces the detachment of endothelial and tumour cells, disorganises the cytoskeleton and the tight junctions, induces apoptosis, and inhibits angiogenesis [98]. A paediatric phase I trial in brain tumours showed similar pharmacokinetics akin to that observed in adults and no dose limiting toxicity [113]. A paediatric phase I trial in combination with irradiation is ongoing for children/adolescents with diffuse brainstem high grade gliomas (CILENT-0902, trial NCT01165333).

### 3.5. Modulation of the Antitumour Immune Response

The immune system may play a major role in EW and OS cancer control. Interestingly, more rapid recovery of absolute lymphocyte count after the very first cycle of chemotherapy is associated with significantly improved survival for both EW and OS [114, 115].

In EW, the proinflammatory microenvironment (interferon, IFN) is more often seen in metastasis than in primary tumours and participates in neoangiogenesis (VEGFR secretion) and the metastatic potential (MMP9 secretion) [116, 117]. The IFN/ifosfamide combination decreases these factors and inhibits tumour growth [116, 117] but at doses that cannot be reached in humans. The intratumour increase in proinflammatory type I cytokines/chemokines correlates with intratumour infiltration by cytotoxic T CD8+ lymphocytes which correlates with tumour progression [118]. *In vivo*, elevated C-reactive protein, a white blood cell count, and profuse vascularisation are associated with tumour macrophage infiltration which correlates with decreased survival [119]. In EW patients, fever is a prognostic factor whatever the metastatic status is [120]. Celecoxib, a COX2 inhibitor, exerts an antiproliferative effect *in vitro* and increases the cisplatin proapoptotic effect [121]. *In vivo*, it prevents pulmonary metastases without any effect on the primary tumour and its vascularisation [122].

The ganglioside, GD2, is expressed on the surface of EW/OS cells [123, 124]. This neuroectodermic marker is targeted by an anti-GD2 monoclonal antibody, which, combined with IL2 and GM-CSF, has significantly increased the survival of metastatic neuroblastoma [125]. One OS patient treated in a phase I trial of ch14.18 had PD [126]. T cells were specifically modified to express the GD2-specific chimeric receptor 14. G2a-28zeta efficiently interacted with EW cells, resulting in antigen-specific secretion of cytokines. Moreover, chimeric receptor gene-modified T cells from healthy donors and from a patient exerted potent, GD2-specific cytolytic responses to allogeneic and autologous EW, including tumour cells grown as multicellular, anchorage-independent spheres. GD2-specific T cells further had activity against EW xenografts [127]. Sargramostim (rhGM-CSF) induces myeloid dendritic cell differentiation facilitating the immune response mediated by T helper lymphocytes. However, the few objective responses were transient [128]. Inhaled sargramostim showed no detectable immunostimulatory effect in pulmonary metastases or improved outcome postrelapse (phase II NCT00066365) [129]. Recently, the identification of the first EFT-specific immunogenic T-cell epitope might lead to a better understanding of EFT immunology and may improve dendritic cell-based immunotherapy [130].

In OS, INF*α*/*β* expression correlates with a better outcome [131], and the presence of infiltrative macrophages is associated with a decreased incidence of metastasis and prolonged survival [132]. IFN*α* induces HLA class I molecule expression and exerts an antiproliferative effect [133]. The results of the randomised combination of IFN*α* with first-line CT in OS (EURAMOS I) are pending. IFN*γ* increases tumour cell surface expression of FAS and lymphocyte T*γδ* cytotoxicity [134]. L-MTP-PE (muramyl tripeptide phosphatidyl ethanolamine liposomal) stimulates the antitumour effect of monocytes/macrophages, facilitates the secretion of proinflammatory cytokines with direct cytotoxic anti-tumour effects (IL1*β*, IL6, TNF*α*) [135], and induces IL12 which destroys circulating OS cells [109]. The US randomised phase III study of L-MTP-PE combined with 1st-line MAP/MAPIE CT in OS (INT-00133) appeared to be in favour of the combination, with a possible positive interaction between L-MTP-PE/ifosfamide [135]. However, the US Food and Drug Administration (FDA) did not approve MTP-PE use in OS, while the European Medicines Agency (EMA) allowed it. OS cells, including chemoresistant variants (doxorubicin, methotrexate, cisplatin), are highly susceptible to lysis by IL-15-induced NK cells of both allogeneic and autologous origin [132].

### 3.6. Modulation of the Bone Microenvironment

Bone tumours are characterized by a vicious cycle between tumour growth and osteolysis, marked by the activity of RANK and its ligand (RANKL), key mediators of osteoclast differentiation, function, and survival [136]. RANKL facilitates osteoclastogenesis, bone resorption, growth factor secretion which participates in bone destruction, tumour growth, and intraosseous migration of RANK+ cells [137]. For OS patients, RANKL tumour expression is associated with a poor response to preoperative CT, high expression with decreased survival, and high TRACP5b plasma levels (osteoclastic activity marker) with the occurrence of metastases [138, 139].

Zoledronic acid, a potent inhibitor of bone resorption by inducing osteoclast apoptosis, also inhibits RANK expression and osteoclast progenitor migration during osteoclastogenesis and increases osteoprotegerin (OPG) expression [140]. In preclinical OS models, it exerts direct antiproliferative [141], proapoptotic/anoikis [142–144], and antiangiogenic effects [145], decreases bone resorption, and exhibits antitumour activity [140, 146–148]. Contradictory data on metastases suggest preventive [143, 148, 149], inexistent [147], or prometastatic effects [150].It overcomes OS resistance to cisplatin [151], irradiation [152], and mTOR inhibitors [145], *in vitro,* and to paclitaxel [140] and ifosfamide [146], *in vivo*. Zoledronic acid combined with 1st-line methotrexate or adriamycin/platinum/ifosfamide-based CT in OS is currently being tested in the French randomised phase III trial (OS2006, NCT00470223). In *in vivo* EW models, zoledronic acid alone is only active against the bone tumour. An effect on extraosseous tumour components is obtained when zoledronic acid is combined with ifosfamide [153]. The use of zoledronic acid in combination with 1st-line chemotherapy is being addressed for localised EW in Europe, in randomised phase III trials (the current Ewing 2008 and future Euro-EWING2012). In juvenile models, zoledronic acid decreases enchondral bone growth in a reversible manner [154]. 

In preclinical OS models, inhibition of RANKL signalling by a decoy receptor OPG or with a soluble form of its membranous receptor RANK (RANK-Fc) inhibits tumour-associated osteolysis and reduces tumour incidence, local growth, invasion, migration, and lung metastases, leading to increased survival in animals [155–157]. However, RANKL inhibition has no effect in OS cells *in vitro* [155–157]. An additive effect of RANKL inhibition with CT was observed in OS models [158]. Fewer data are available in EW, but indirect RANKL inhibition leads to inhibition of osteoclastic activity [159, 160].

Denosumab is a humanised monoclonal antibody (IgG2) with high affinity and specificity against RANKL and is interesting in several cancers with bone metastases [161]. A phase II safety study of denosumab in subjects ≥12 years with a recurrent/unresectable bone giant cell tumour is ongoing (NCT00680992) [162].

In addition to the antiangiogenic effects, DDK1 inhibition (Wnt pathway) by the monoclonal antibody BHQ880 might restore bone formation but without a direct antitumour effect. BHQ880 is currently being investigated in adult phase I/II trials for multiple myeloma, alone (NCT01302886; NCT01337752) or associated with zoledronic acid (NCT00741377).

Bone-seeking radiopharmaceuticals provide another bone-specific means to target OS cells, which make bone. The standard 99mTc-MDP bone scan is the screening test of this characteristic needed for targeting. The beta emitting 153Sm-EDTMP (Samarium) is FDA approved for osteoblastic bone metastases and is useful for palliation of pain. A newer alpha-emitting radiopharmaceutical, 223Ra (Alpharadin), may be not only more safe (less marrow toxicity) but also more effective because the dense energy deposited by alpha particles produce double strand breaks [163–166].

### 3.7. Other Exploitable Therapeutic Pathways

#### 3.7.1. Hedgehog Pathway Inhibitors (SHH/PATCH/Smo/GLI)

The Hedgehog signalling pathway plays an key role in growing organisms (embryogenesis, morphogenesis) and is activated in OS/EW (GLI is an EWS-FLI1 target) [167, 168]. Its inhibition by cyclopamine in OS [169] and arsenic trioxide, a GLI inhibitor, in EW [168], stunts tumour growth. Arsenic trioxide reverts multi-CT resistance in OS cell lines [170]. A paediatric phase I study is ongoing testing LDE225, a smoothened inhibitor (NCT01125800). Its effects on bone growth might be of concern. Another inhibitor of this pathway is Itraconazole at an antifungal dose [171].

#### 3.7.2. Histone Deacetylase (HDAC) Inhibitors

HDAC and histone acetyltransferase (HAT) are enzymes which catalyse histone deacetylation and acetylation, respectively, and modify chromatin access to transcription factors and gene transcription. Two paediatric phase I trials have been completed with two HDAC inhibitors (vorinostat and valproic acid) [172, 173]. 

In OS models, HDAC inhibitors decrease DNA repair capacity [174], sensitising cells to irradiation [175] and doxorubicin [176, 177], facilitate Fas-dependent cell death by increasing Fas expression on tumour cells which die through apoptosis in the presence of FasL (lung) [178], and decrease FLIP expression, a negative regulator of caspase 8 [179]. SNDX-275 nasal administration exerts a preventive action against pulmonary metastases in murine OS models [178]. Valproic acid increases membrane HLA class I molecule expression, sensitizing OS cells to NK cytotoxicity [180]. HDAC inhibitors are suspected of negative effects in OS, through the induced expression of Notch genes and invasion, which might facilitate the OS metastatic potential [99].

In EW cells, EWS-FLI1 represses HAT and activates HDAC [181]. HDAC inhibition restores HAT activity, inhibits cell growth, and induces apoptosis [182]. FK228 decreases EWS-FLI1 expression and EW proliferation [181] and induces TRAIL-dependent apoptosis [183].

Acquired resistance to the cyclic tetrapeptide family HDAC inhibitor (FK228) is mediated by P glycoprotein (PgP), a drug efflux pump and the MAPK pathway, and might be reverted with verapamil (EW) [184] and MEK inhibitors (OS) [185].

#### 3.7.3. Heat Shock Protein 90 (HSP90) Inhibitors

HSP90 is a chaperone protein implicated in numerous cancers. It is overexpressed in 21/54 EW patient samples [186]. Anti-HSP90 antibodies in sera are associated with a poor response to CT in OS [84].

HSP90 inhibitors induce proteasome-mediated degradation of many oncogenic proteins involved in all hallmark characteristics of cancer. 17-AAG induces *in vitro* apoptosis [29] and *in vivo* tumour growth retardation in OS as a single agent and in combination with cisplatin [187] and restores the efficacy of the IGF1R inhibitor and imatinib in EW models [186]. No objective response was observed in two paediatric phase I trials (SD 1/3 EW, 0/7 OS). However, acquired resistance to 17-AAG is rapid [188], and new generations of HSP90 inhibitors might be more promising (adult phase I/II trials ongoing).

## 4. Conclusion

The multiplicity of targets in primary malignant bone tumours in children/adolescents, the increasing number of new molecular therapies becoming available, and the rarity of these tumours will not allow testing all of the strategies which are discussed in this paper. Consequently, prioritisation in drug development as well as new methodologies for the development of therapeutic trials will be required.

In EW, the development of therapies targeting the EWS-FLI founder genetic abnormalities is crucial, but currently at an extremely early stage. The experience with anti-IGF1R antibodies suggests that the inhibition of EWS-FLI targets might be useful to control the disease in some patients but not in a prolonged manner if used as monotherapy. Combination with CT should be tested, and a better understanding of the predictive factors of response is compulsory. In addition, due to the multiplicity of EWS-FLI targets and the pathways redundancies, simultaneous inhibition of growth factor receptor and downstream pathways might be useful to overcome some resistance, as well as, targeting different characteristics of the tumour and the environment such as bone microenvironment (Zometa phase III), angiogenesis (bevacizumab phase II), and antitumoural immunity (anti-GD2 humoral or cellular immunity).

In OS, no founder mutation is known and more efforts are necessary to understand the biological processes implicated in OS oncogenesis. Strategies targeting antitumoural immunity (MTP-PE, phase III first-line trial), angiogenesis (sorafenib, phase II trial), and bone microenvironment (zoledronic acid, preclinical data) appear promising, including in association with cytotoxic CT. Combining these strategies together and with first-line CT as well as developing therapies directed against the metastatic process (e.g., MET inhibitors) might further improve OS outcome. In conclusion, future therapeutic strategies against bone tumours will reside in the way we combine therapies targeting different characteristics of the malignant cells and their environment.

## Figures and Tables

**Table 1:** Molecular targets according to malignant characteristics and current development of targeted therapies in osteosarcomas and Ewing sarcomas.

Targets	Agents	Clinical development in different tumour types	Osteosarcoma	Ewing sarcoma
EWS-FLI1 inhibition	Antisense oligonucleotide, antisense RNA, siRNA			P
	Mithramycin			I ped/II ad ongoing, NCT01610570
	YK-4-279			P
	ET-743 (trabectedin; Yondelis*)		I ped/II ad	I ped/II ped

Cell growth Inhibition				
GFR inhibitors				
IGFR inhibitors	R1507; SCH 717454; CP-751871; IMC-A12		I ped	II ped (EW: 10–15% objective response rate)
mTOR inhibitors	Everolimus (RAD001, Afinitor*)		I/II ped	I ped
	Temsirolimus (Torisel*)		I/II ped	I/II ped
	Ridaforolimus	I ped ongoing, NCT01431547 all solid tumours	II/III ad	II/III ad
Combination	EWS-FLI1 antisense oligonucleotide + mTOR inhibitor		/	P
	Rapamycin + irinotecan (RAPIRI)	I ped ongoing, NCT01282697 all solid tumours	P	P
	Cixutumumab + temsirolimus		II ped/II ad ongoing (NCT01614795)/	II ped/II ad ongoing (NCT01614795)
	Ridaforolimus + dalotuzumab	I ped ongoing, NCT01431547 all solid tumours	I ped	/
Multitarget inhibitors	Imatinib mesylate, Glivec* (PDFGR, c-KIT, BCR-ABL)		P	II ped
	Imatinib + ifosfamide		I ped	/
	Dasatinib, Sprycel* (Src, BCR-ABL)		P	I ped
Cell cycle inhibitors	CDK inhibitors SCH 727965 (dinaciclib)		I ad	/
	Rexin-G			/
	Aurora A inhibitors		P	
	MLN8237	I/II ped ongoing, NCT01154816/NCT00739427		P
		Solid tumours or leukemia	P	
	AT9283	I ped ongoing, NCT00985868/NCT01431664		P
		Solid tumours/leukemia	P	
	PLK1 inhibitor, BI 2536		P	/
	MDM2 inhibitors, nutlin-3	I ad ongoing, NCT01462175 solid tumours		P

Angiogenesis inhibition	Sorafenib, Nexavar* (Raf, c-KIT, PDGFR, VEGF)		II ad	I ped ongoing, NCT01518413
	Sunitinib, Sutent* (Flt3, c-KIT, PDGFR, VEGF)		I ped	I ped
	Pazopanib (VEGFR1–3, PDGFR*α*/*β*, c-Kit)		P	I ped
	Pazopanib + topotecan	I ped ongoing, NCT00326664 CNS tumours	P	P
	Pazopanib + everolimus	I ad ongoing, NCT01430572 solid tumours	/	/
	Cediranib, AZD2171 (VEGFR)		III ped/ad	I ped
	Bevacizumab Avastin* NCT00667342 trial		I/II ped	I/II ped
	Bevacizumab + vincristine/topotecan/cyclophosphamide		/	II ped ongoing, NCT00516295
			III ped/ad ongoing, NCT00667342	/
	Bevacizumab + CT		1st-line randomised study, combination CT	

Resistance to cell death				
Apoptosis	BCL2 inhibitors, navitoclax (ABT-263)		/	P
	TRAIL inhibitors		P	P
	SMAC mimetic, LCL161	I ad ongoing, NCT01098838 solid tumours	P	P
	PARP inhibitors		/	P
Autophagy	Antisense oligonucleotide of X-linked IAP		P	P
Telomerase activity	Telomerase inhibitor, TMPyP4		P	P

Inhibition of metastatic phenotype				
Invasion	Gamma secretase (NOCTH Inhibitors), MK-0752	I ad/ped leukemia	P	P
Migration	MET/ALK inhibitors (Crizotinib)	II ped >15 y ongoing, NCT00939770, solid tumours	P	/
Resistance to anoikis	GSK3beta inhibitors (Wnt pathway activation)	I ad ongoing, NCT01457417, NCT00741377 myeloma	P	/
Chemotactism	CXCR4 inhibitors (plerixafor)	I ped ongoing, NCT01319864 leukemia/MDS	P	P
Adhesion	Integrin inhibitors, cilengitide EMD121974	I ped ongoing, NCT01165333 CNS	P	/

Modulation antitumour immune response	INF*α*		III ped/ad (EURAMOS I trial, results awaited)	/
			1st-line randomised study, combination CT	
	L-MTP-PE, mifamurtide MEPACT*		III ped/ad (INT-0133 trial closed)	/
	Inhaled sargramostim (rhGM-CSF)		II ad ongoing, NCT00066365: Pulmonary relapses	/
	Celecoxib, COX2 inhibitors		/	P
	Anti-GD2 antibodies (ch14.18)	I/II ped, neuroblastoma	I ped	P

Bone microenvironment			III ped/ad OS2006 trial ongoing, NCT00470223	III ped/ad protocol ewing 2008 and EE2012
	Zoledronic acid, Zometa*		1st-line randomised study, combination CT	1st-line randomised study, combination CT
				For localised EW + good histological response
	Denosumab (Ac anti-RANKL)	II ad/ped >12 ans ongoing, NCT00680992 GCT	P	P
	Samarium		II ad	/

Other pathways				
Hedgehog inhibitors	Smo inhibitor LDE225 (ongoing)	I ped ongoing, NCT01125800 solid tumours	P/I ped, NCT01125800	P
HDAC inhibitors	Vorinostat, valproic acid, FK228		P/I ped	I ped
HSP90 inhibitors	17-AAG		I ped	I ped

CNS: central nervous system; GFR: growth factor receptor; P: preclinical studies; I: phase Itrial; II: phase IItrial; III: phase IIItrial; ped: paediatric; ad: adult; CT: chemotherapy; CGT: giant cell tumour.

## Abbreviations

CR: Complete response
CT: Chemotherapy
EFS: Event-free survival
EMA: European Medicines Agency
EW: Ewing sarcoma
FDA: The US Food and Drug Administration
HAT: Histone acetyltransferase
HDAC: Histone deacetylase
IAP: Inhibitor of apoptosis proteins
IGF-1R: Insulin-like-growth-factor 1 receptor
OPG: Osteoprotegerin
OS: Osteosarcoma
PD: Progressive disease
PFS: Progression free survival
PPTP: Pediatric Preclinical Testing Program
PR: Partial response
RANK: Receptor activator of nuclear factor-*κ*B
RANKL: RANK ligand
SD: Stable disease
SFCE: Société Française des Cancers de l'Enfant
siRNA: Small interference RNA.
